# Barriers to and Facilitators of School Health Care for Students with Chronic Disease as Perceived by Their Parents: A Mixed Systematic Review

**DOI:** 10.3390/healthcare8040506

**Published:** 2020-11-21

**Authors:** Ju-Yeon Uhm, Mi-Young Choi

**Affiliations:** 1 Department of Nursing, Pukyong National University, Busan 48513, Korea; jyuhm@pknu.ac.kr; 2 Department of Nursing Science, Chungbuk National University, Cheongju 28644, Korea

**Keywords:** adolescent, caregivers, child, child health, chronic disease, healthcare review, school nursing

## Abstract

Understanding parental perspectives through mixed systematic reviews is imperative for developing effective school health care for children and adolescents with chronic disease. A mixed systematic review was conducted to explore barriers to and facilitators of school health care for students with chronic disease as perceived by their parents. Four databases (2010–2020) were searched, following which critical appraisals were conducted to determine the validity of the selected studies using the Mixed Method Appraisal Tool, version 2018. Twenty articles were synthesized using the convergent integrated approach from the Joanna Briggs Institute’s mixed method systematic review methodology. We examined 20 articles regarding parents’ perceived barriers and facilitators and found views across four levels: intrapersonal, interpersonal, institutional, and public and policy. Parents perceived more barriers than facilitators. Barriers on the institutional level were the most frequently reported of all levels of barriers. These results suggest that multi-level school health interventions could be a valuable resource to facilitate effective school guidelines and public policies for students with chronic diseases.

## 1. Introduction

For children under the age of eight, the prevalence of asthma (8.5%), epilepsy (0.69%), and diabetes (0.5%) is a concerning matter [1]. From 2009 and 2014, chronic conditions accounted for more than 60% of hospital readmissions for children and adolescents aged 1–17 years in the US [2]. From the ages of 15 to 30 years, the rates of patients with type 1 diabetes mellitus (T1DM) for 30-day unplanned hospital readmissions increase significantly, with the odds of readmission peaking at the age of 23 years [3].

Chronic disease in children affects their—and their families’—whole lives. As such, children are significantly influenced by their parents and cannot be examined separately from their parents; therefore, child health care should be considered within the family context. Parenting factors such as stress and parent-child interaction are associated with the outcomes for children with chronic disease [4]. Accordingly, understanding the needs and experiences of parents of children with chronic conditions is critical to develop effective collaborative practices [5]. Thus, in health care for children with chronic disease, practitioners should focus on both parents and their children.

As children and adolescents spend a significant amount of time at school, most parents recognize that school health care (SHC) is important for managing their child’s health. They positively perceive that the school-based educational program provides correct information about diseases such as T1DM [6]. Nevertheless, parents are concerned about how their child will adapt to school life, improve attitudes, relieve symptoms, and establish self-management abilities [7,8]. They also fear complications and inappropriate medication in school [6]. Specifically, parents of younger children have significant fears about potential emergencies occurring when their children are at school [9].

Accordingly, parents with chronically ill children and adolescents have reported that the current SHC is inadequate, and some asserted the need for more education and counseling in schools [6,10,11]. Moreover, parents expected schools to manage emergencies effectively [10,12] and have a clear care plan [11].

SHC for students with chronic diseases requires more cooperation between parents and school professionals [5] as school nurses have reported that there is currently limited collaboration [13]. Thus, it is crucial that schools engage with both students and their parents to assess the risk of chronic disease in SHC [14]. The understanding of parental perspectives gained from a mixed systematic review is imperative for developing effective SHC for students with chronic diseases.

Therefore, the aim of this study was to conduct a mixed systematic review on the perception of parents of students who are chronically ill regarding SHC. To the best of our knowledge, no such reviews exist in the literature. The results of this study may provide a foundation for developing an SHC strategy reflecting parental perspectives for improvement.

## 2. Literature Review

### 2.1. Design

A mixed systematic review was conducted based on the convergent integrated approach of the Joanna Briggs Institute’s mixed method systematic review methodology and stages [15]. A mixed systemic review is divided into convergent and sequential approaches, depending on whether synthesis occurs simultaneously or consecutively [16]. While a systematic review synthesizes the results of quantitative or qualitative studies, mixed review studies—also called mixed study reviews—synthesize quantitative, qualitative, and mixed studies to fill the gaps of each study type [17]. The mixed-methods model provides readers “quantitative estimates of benefit and harm” or “facilitators and barriers,” in addition to an understanding of qualitative perspectives or experiences in specific contexts [15,18].

### 2.2. Review Question

This study aimed to identify and synthesize knowledge about the barriers and facilitators of SHC for students with chronic disease, as perceived by their parents. The review question was “what do parents of children with chronic disease view as the barriers and facilitators in SHC?”

### 2.3. Inclusion Criteria

According to the review question, PICo (population, phenomena of interest, and context) has been defined. This review considered studies that included parents of children with chronic disease as the population, perceived barriers and facilitators of caring as the phenomena of interest, and SHC as the context. This study defines children with chronic diseases as those aged 3–19 with chronic health issues, such as asthma and diabetes, who require regular hospital care, medication, and self-regulation. This definition is based on the covered age range used in laws such as the Individuals with Disabilities Education Act and Section 504 of the Rehabilitation Act of 1973 for providing school health services [19], and the definition used in a prior systematic review [20].

### 2.4. Search Strategies

A systematic search using PubMed, Cumulative Index to Nursing and Allied Health Literature (CINAHL), Embase, and Web of Science (WOS) was performed to identify studies published between January 2010 and June 2020 that met the inclusion criteria (i.e., written in English, peer-reviewed, and primary articles). The search strategy included keywords that incorporated five contexts: (1) barriers and facilitators, (2) parents, (3) children and adolescents, (4) chronic disease, and (5) school context. We also developed the final search terms using synonyms. Keywords and synonyms were applied in the “Title/Abstract” field for the PubMed and Embase databases. Keywords and synonyms were applied in the “TS (topic), TI (title), and ALL” strategy in the Web of Science database and the “MW (Word in subject heading) and all filed” strategy in the CINAHL database. Boolean operators “OR” and “AND” were used.

### 2.5. Study Selection

**Figure 1:**
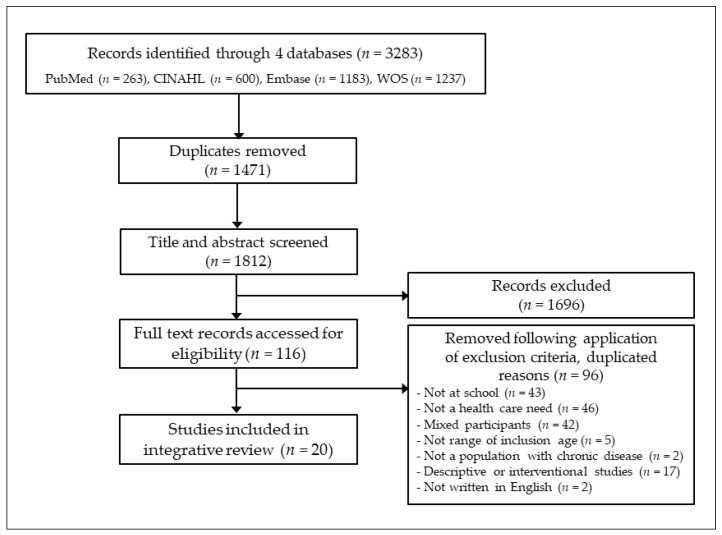
Preferred Reporting Items for Systematic Reviews and Meta-analyses (PRISMA) flow diagram was applied [21], with the flow diagram showing the search process. CINAHL, Cumulative Index to Nursing and Allied Health Literature; WOS, Web of Science.

All identified citations were loaded into Endnote; duplicates were removed. Titles and abstracts were screened by two independent authors for studies fulfilling the inclusion criteria. Lists of potentially relevant studies were recorded in a spreadsheet. The full texts of potentially selected studies were reviewed in detail against the inclusion criteria by two independent reviewers. The texts were then checked for exclusion criteria (Figure 1) and 20 articles were included in this review.

### 2.6. Assessment of Methodological Quality

Critical appraisals were conducted to determine the validity of the selected studies. Two authors independently used the Mixed Method Appraisal Tool (MMAT), version 2018 [22], to appraise the selected articles’ methodological quality. Six qualitative studies, 12 quantitative studies, and two mixed studies were appraised using the tool, which identified five, five, and 15 items, respectively. Quantitative studies had an MMAT rating of 100% each, while one of the qualitative studies’ rating was 80% and the ratings of two mixed methods studies were evaluated as 73.3% and 86.7%. The evaluation results are presented in the Supplementary Material (Table S1).

### 2.7. Data Extraction and Transformation

We used a convergent integrated approach for data extraction. In quantitative studies, data extraction includes all relationships relevant to the review question and significant and non-significant results. For qualitative studies, categories and subcategories related to the review question were extracted through a direct quotation or other contextual data. Quantitative data were transformed into so-called “qualitized data” [15]. Conversion into “qualitized data” is the transformation of quantitative data into narrative findings with descriptive statistics that respond to the review question.

### 2.8. Data Synthesis and Integration

A convergent integrated approach involves content analysis, vote count, and thematic analysis for integration [15]. Extracted and transformed data were integrated using content analysis [23]. Two authors identified codes, grouped codes to develop potential subcategories, and generated subcategories. In this review, the headings of the main categories were defined using the ecological model [24] as an explanatory model. General categories and subcategories were synthesized based on the four levels of analysis of the ecological model and we checked the validation of the data synthesis.

### 2.9. Conceptual Framework

The synthesis of this mixed systematic review adopted the ecology perspective for health promotion, based on Bronfenbrenner’s model as a conceptual framework [24]. The ecological model of health behavior was applied to explain behavior and guide behavioral interventions [25]. This model was explained using five levels, namely intrapersonal factors, interpersonal processes and primary groups, institutional factors, community factors, and public policy [24]. This model can be useful for understanding the multifaceted and interactive views of families’ characteristics and school health-related environmental factors [26]. In this model [24], the intrapersonal level included individual factors such as knowledge, attitudes, behavior, self-concept, and skills. The interpersonal level included social relationships, which influence individual health related behaviors, such as family or contacts at work. The institutional level included social institutions with organizational characteristics and rules and regulations for operation. The community level included relationships among organizations, institutions, and informal networks; the public level included local, state, and national laws and policies. In this review, intrapersonal and institutional factors were adopted and approached the same way they had been in previous studies [24,25]. The interpersonal level was defined as relationships with stakeholders related to school health, including the provision of social resources such as support, information, tangible aid, and assistance for families by relieving some of the burden of managing the child’s health and supporting the child in learning self-management. The community level was merged with the public policy level. Based on this evidence, the ecological model was used to understand parents’ perceptions of the barriers to and facilitators of SHC for students with chronic diseases.

## 3. Results

Twenty articles met the review’s inclusion criteria. There were six qualitative and 11 quantitative studies, and three were conducted using a mixed method. There were 12 cases of T1DM, six of asthma, one of a food allergy, and one of various chronic diseases. We summarize the selected articles’ findings in the Supplementary Material. A synthesis of the key findings identified barriers and facilitators on four levels (Table 1 and Table S2).

### 3.1. Intrapersonal Level

#### 3.1.1. Intrapersonal Barriers

Parents perceived barriers related to the lack of knowledge and awareness of school staff in five articles and barriers related to their own lack of knowledge in three articles. Parents also pointed out a lack of awareness about chronic disease among school staff [11,27,28,29] and lack of sufficient sensitivity regarding undesirable events among teachers and school leaders [28]. Some reported that the practical difficulties in daily management were related to lack of knowledge and awareness among school staff [12].

Parents had difficulty in completing paperwork for reporting and verification at school [30]. There was a lack of knowledge about managing disease among parents, despite SHC [31]. Most parents (74%) reported lack of awareness about the diabetes school’s information resource [32].

#### 3.1.2. Intrapersonal Facilitators

Parents perceived that school-based interventions and action plans can facilitate parental knowledge. They were satisfied with a school-based intervention for improving knowledge [31] and noted that clear action plans can increase the level of awareness in the school [11].

### 3.2. Interpersonal Level

#### 3.2.1. Interpersonal Barriers

In eight articles, parents perceived barriers to SHC on an interpersonal level, including limited communication between school and family, lack of collaboration with school professionals, and difficulties in peer relationships.

Parents identified barriers regarding limited school-family communication [11,28,30,33]. They also needed communication with the school regarding any changes to the care plan [34]. In addition, there was a lack of collaboration among school staff. Two-thirds of the parents perceived that schools were not prepared to address families’ needs and 67% experienced conflicts with school professionals in treating T1DM [6]. Parents perceived difficulties in peer relationships and had concerns about their children’s relationships with peers due to their conditions [6,7,33].

#### 3.2.2. Interpersonal Facilitators

In six articles, parents perceived facilitators of SHC on the interpersonal level, such as effective communication between school and family, parental engagement, collaboration with school professionals, clear role delineation, and school-based interventions. Parents believed that direct parent-teacher communication can facilitate SHC [11]. Parents perceived the importance of parental engagement regarding their children [34] and were involved in or made decisions for all children’s health conditions, besides facilitating their child’s SHC [11,12,32,34,35].

Parents perceived collaboration with school professionals and a clear role delineation as a facilitator of SHC [29]. Parents collaborated with school professionals on assisting children’s adaptation into school by providing information about handling emergencies and they perceived communication with teachers positively [7]. Parents wanted a clear role delineation among parents, school nurses, and primary caregivers for specific portions of SHC [34].

Parents believed that school-based interventions can facilitate SHC. Some parents responded that face-to-face meetings through a school-based project can improve communication in SHC [34]. They were satisfied with a school-based intervention for improving the relationships among their child, school staff, and classmates [6].

### 3.3. Institutional Level

#### 3.3.1. Institutional Barriers

In 15 articles, perceived barriers to SHC on the institutional level included limited school guidelines, school staff related barriers, unsafe school environment, limited self-care support, insufficient services for parents, and insufficient advocacy. Parents reported limited school guidelines from health care providers for managing diseases [11,27,28] and that their children felt embarrassed at school [27].

Parents perceived various school staff-related barriers. First, parents reported inadequate staffing and no disease point persons to support their child’s health care. For example, there was no disease point person to administer insulin during school [12] or any person responsible for managing diabetes in the school [27]. Parents had concerns regarding lack of school nurses and anxiety about unpredictable life-threatening events [30,36,37]. Second, parents perceived lack of staff education and training, such as for glucagon injections [27] and in managing diabetes [29,32]. They were concerned about qualified staffing and retraining of new teachers or staff [9]. Third, parents perceived unclear roles in terms of the responsibility among school staff. Parents of children with a food allergy reported that a diverse school staff carries epinephrine on field trips and after-school activities and travels with every group [38]. Parents experienced lack of teacher involvement [36] in managing diseases.

Parents reported various aspects of an unsafe school environment. First, they perceived limited support for medication administering, particularly for using inhalers and administering insulin [9,33]. Parents reported the occurrence of incorrect insulin administration [37]. Second, parents perceived a lack of confidence among teachers regarding emergency response procedures [7,9,33], lack of equipment such as a refrigerator to store glucagon [12], and limited availability of emergency medication [27,38]. Third, they perceived a limited allergen-free environment. They were concerned about school-based asthma triggers [33]. Parents of children with food allergies perceived schools as unsafe or were unsure [38]. Allergen information about lunch menus or each food items was often unavailable [38]. Fourth, parents reported difficulties in maintaining stable blood glucose levels. Most parents of children with T1DM reported their children had experienced at least one hypoglycemic event during school hours [12,29]. They were also concerned about extreme blood glucose levels and administering/adjustment of insulin in school [6,9,35]. Fifth, parents perceived inadequate lunch services for children with T1DM [27,36] and had concerns about diet supervision [9] and food during excursions [35].

Parents perceived limited self-care support [11] and reported unavailability of educational material about food allergies [38].

Parents perceived insufficient services for themselves, including limited parental education by school nurses [39] and a lack of access to information about managing disease [28,29,33].

Finally, parents perceived insufficient advocacy for participation in school activities. They stated that students with diabetes were often withdrawn from classroom activities [6].

#### 3.3.2. Institutional Facilitators

Eleven articles described parents’ perceived facilitators of SHC on an institutional level, including school staff-related facilitators, safe school environments, supported self-care, services for parents, and a tight-knit community.

Parents perceived various school staff-related facilitators. First, they perceived sufficient staffing and a disease point person as a facilitator. Parents expected a full-time, trained disease point person in SHC [11] and were significantly satisfied with self-management in school when such a point person existed [35]. Sufficient nurse staffing was correlated with diabetes-related safety and satisfaction [39]. Second, they perceived school staff education and training as a facilitator of SHC [11,33,36]. Parents wanted there to be training for coaches/gym teachers, as well as school nurses [37].

Parents perceived a safe school environment as a facilitator and identified first aid stations as facilitators of SHC [6]. Parents who reported the ability to check blood glucose levels conveniently were 19.6 times more likely to be satisfied with their child’s care at school [36].

Parents perceived supported self-care as a facilitator and that the school should help their child with independent self-care [33], which is a major facilitator [11]. School nurses’ helpfulness was correlated with diabetes-related safety and satisfaction [39]. Parents reported an increase in children’s abilities to self-manage and keep up with school work because of school nurses’ case management [40,41]. They were also satisfied with a school-based intervention for self-management [31].

Parents also reported that services for parents, such as parental education and access to more information, help facilitate SHC. Some parents needed education about disease or SHC, including parents of students without disease [33]. They needed access to more information regarding their child’s health, but were satisfied with sharing information on health through an educational project on diabetes. Parents were also satisfied with the behavior of primary caregivers and professionals in the school [6]. They perceived that primary caregivers and school nurses should further educate parents about topics such as asthma attacks [34].

Parents regarded a tight-knit community as a facilitator of SHC. Parents needed education about diseases or SHC, including for students without disease [33]. Parents of children with asthma perceived that school-based education for children and peers could prevent delays in treatment and continuation of physical activity despite symptoms; they also believed that senior peers can assist in emergencies [11]. They desired mandatory school-based education to encourage a tight-knit community and sharing information about children with chronic disease among all school staff [11].

### 3.4. Public and Policy Level

#### 3.4.1. Public and Policy Barriers

In six articles, parents perceived barriers to SHC, including limited school policy, lack of coordination between education and health care systems, inequity of SHC, and inaccessibility to schools. Parents perceived limited policies to be a barrier to maintaining a safe school environment [28,38]. They identified lacking a formal plan—such as Asthma Action Plans or Section 504 plans—as a major barrier to SHC [11]. There were inequities between the school policies of private and public schools [27,38] and disparities between minority and white students [36]. Parents reported a lack of coordination between education and health systems [27,28]. They had difficulties in contacting treating physicians during school hours [27] and felt there was limited coordination between school, family, and health providers [28]. One barrier included inaccessibility to school due to work and transportation [32].

#### 3.4.2. Public and Policy Facilitators

In three articles, parents perceived facilitators to SHC at the public and policy level, including clear action plans and legal support for school health policies [11,35]. Parents were satisfied with a written action plan for the treatment of hypoglycemia [35]. They identified legislation such as the US’s Asthma Action Plan or Section 504 plan as being facilitators of SHC [11]. Parents recommended that stakeholders should be involved in developing school health policy [33].

## 4. Discussion

This mixed systematic review used the ecological model to identify barriers to and facilitators of SHC on four levels from the perspective of parents with children suffering from chronic diseases. The ecological model emphasizes the significance of both individual and social environmental factors for health promotion [24]. Parents perceived barriers and facilitators on multiple levels; additionally, they perceived more barriers than facilitators. In particular, barriers at the institutional level were the most frequently reported of all levels of barriers. Schools and policies may restrict or foster children’s and family’s self-management in school, as well as individual motivation or effort [42]. The order of the four levels of categories derived from the results—intrapersonal, interpersonal, institutional, and public policy level—are discussed below.

First, parents perceived barriers on the intrapersonal level, such as lack of knowledge and awareness among school staff. Lack of knowledge or confidence is in line with a lack of school policies and guidelines, which results in a lack of staff education and training at the institutional level. Further, a lack of knowledge is a barrier to chronic disease management, as identified by school nurses [43]. More tailored training opportunities are needed as lack of time is a barrier to school nurses’ ability to provide comprehensive SHC [44]. Multimedia education can facilitate increased knowledge and self-efficacy among parents of children with chronic disease [45]. Moreover, based on the ecological model, each level’s influences interact across other levels [25]. Accordingly, educational support is needed for the institutional and public levels.

Second, coordination of school health; a school-parent partnership; and collaboration among clinicians, school nurses, families, and community are essential to facilitate SHC. School nurses play a key role in school-family communication. However, limited resources, including lack of time and staff, hinders effective communication [43]. Thus, it is necessary to identify the communication gaps between school nurses and parents about SHC and find ways to communicate effectively [46]. Clear role delineation is also necessary for effective collaborations for managing chronic disease and collaboration among stakeholders is needed to improve the quality of SHC [47]. Parental engagement was one of the facilitators of SHC. This finding is in line with the positive effects of parental involvement in SHC, such as preventing unhealthy behaviors [48]. The CDC (2013a) suggested a provision of parental support, encouraging participation in decision making and communication to foster parental engagement.

Third, parents recognized barriers and facilitating factors for qualitative preparation for health care in schools. The most important generic categories in an institution were the staff and a safe school environment, including factors to effectively manage asthma attacks, prevent sudden complications, and proficiently cope with emergencies, such as cases of anaphylaxis. In one review, adolescents with T1DM and their parents perceived a lack of full-time school nurses and teacher knowledge about diabetes [49]. Outside the classroom, including the playground, field trips, and after-class activities, parents were able to recognize the preparedness of all school staff. Young children with T1DM often have difficulty with glucose testing and they may not receive adequate insulin therapy. An insulin pump is an easy, comfortable method for administering insulin, but 34% of primary students still need self-injection [50]. This situation is a good example of the need for a disease point person for convenient testing and medication safety.

Building a school environment of medication safety and convenience, with emergency response procedures in place, was a significant factor of qualified SHC. The medication administering error rate was 15.3% for a year in one study and the mean number of errors was 1.2 for licensed health care personnel and 7.4 for unlicensed assistive personnel [51]. This study shows the significance of staff training and a supportive atmosphere for medication safety. Parents reported the importance of setting up emergency response procedures. However, the availability of emergency equipment such as ambu-bags and oxygen were low, at 27% and 11%, respectively [52]. Parents also perceived the need for allergen-free environments, convenience for testing, and adequate nutritional services. The CDC recommended that children and parents should be educated and a safe school environment should be created [53].

This review revealed that parents expect schools to strengthen and support children’s self-management. School-based intervention can improve children’s self-management knowledge and skills related to asthma self-management [54]. Stakeholders should promote competency and autonomy to support self-management in children with chronic disease, and children’s developmental trajectory should be considered by families and health care professionals in promoting self-management [55]. Interventions within the family are essential to maximizing children’s self-management abilities. Motivating parents can facilitate their children’s caregiving.

Parents reportedly perceived a need for more education and more access to information. Educational strategies using the Internet [56] or peer coaching interventions [57] could increase satisfaction for parents of children with chronic disease. These strategies to provide services for parents can be included when developing SHC. Parents also wanted their children to experience routine school life without discrimination. To facilitate normalcy for the children, cautious prevention and monitoring of complications is needed, rather than sending children home if they have a chronic health-related event. Parents perceived developing a tight-knit community in the school as a facilitator. Peer mentors can provide informational, appraisal, and emotional support for adolescents with chronic disease [58]. Parents desired a tight-knit community, as they wanted the surrounding people to be able to immediately help their children in emergencies. To develop such cohesive and cooperative communities, educational projects are needed to increase awareness of chronic diseases.

Fourth, parents perceived the importance of policy for SHC. Environmental and policy affect health inequities [42]. In the US, children with chronic diseases are protected under laws such as Section 504 of the Rehabilitation Act of 1973, Americans with Disabilities Act, Individuals with Disabilities Education Act [59], and Asthma Action Plan. However, parents of children with T1DM, asthma, and food allergies still need robust and clear, understandable SHC systems through legislation and policies. Stakeholders desire the confirmation that there are no legal problems when providing SHC for children with chronic diseases. School nurses’ confidence regarding emergencies was related to the presence of medical emergency response plans [52]. The presence of laws and policies is significant to ensure school nurses’ ability to facilitate SHC for students with chronic disease [60]. Continuous administrative support is needed for systematic approaches to SHC. Stakeholders should make efforts to become policy advocates for students with chronic disease [44].

### Limitations

In this review, the types of chronic diseases presented in the included studies are biased in specific areas and culturally biased results may have been derived. The methodological complexity of a mixed method review can lead to reduced rigor and inaccuracy.

In addition, the grades or ages of children in the selected articles had wide ranges. Although the main group of students covered by this review could have been of elementary school age, we aimed to include a wider age group to encompass more than two developmental stages of children. Future studies should examine the perceptions of parents with children of specific grades or age ranges or compare them according to age ranges.

## 5. Conclusions

This study used a mixed systematic review method to explore parental perception regarding SHC for students with chronic disease. We examined 20 relevant articles and found barriers to and facilitators of SHC on intrapersonal, interpersonal, institutional, and public policy levels. These results suggest that multi-level school health interventions could be a valuable resource to facilitate effective school guidelines and public policies to build a safe school environment for the students with chronic disease.

## Figures and Tables

**Table 1:** Synthesis of the key findings.

Main Category	Generic Categories and Subcategories	
	Barriers	Facilitators
Intrapersonal level	*Lack of knowledge and awareness of school staff* *Lack of parental knowledge*	*School-based intervention* *Action plan*
Interpersonal level	*Limited communication between school and family* *Lack of collaboration with school professionals* *Difficulties in peer relationships*	*Effective communication between school and family* *Parental engagement* *Collaboration with school professionals* *Clear role delineation* *School-based intervention* Parental education by school nurse
Institutional level	*Limited school guidelines* *School staff-related barriers* Insufficient staffing and no existence of disease point person Lack of staff education and training Unclear role responsibility *Unsafe school environment* Limited medication administering support Unconfident emergency response procedure Lack of equipment Limited availability of emergency medication Limited allergen-free environment Difficulties in maintaining stable blood glucose level Inadequate lunch services *Limited self-care support* *Insufficient services for parents* Limited parental education and access to information *Insufficient advocacy* Discrimination in all school activities	*School staff-related facilitators* Sufficient staffing and disease point person Staff education and training *Safe school environment* A first-aid station Convenient testing *Supported self-care* *Services for parents* Parental education and access to more information *Tight-knit community*
Public and policy level	*Limited school policy* *Lack of coordination between the educational and health care systems* *Inequity* *of school health care and inaccessibility to school*	*Clear action plan and legal support for school health policy*

The italicized text refers to generic categories that also include subcategories.

## Supplementary Materials

The following are available online at https://www.mdpi.com/2227-9032/8/4/506/s1, Table S1: Critical appraisal of potentially selected articles, Table S2: Summary of the included studies. References [6,7,9,11,12,27,28,29,30,31,32,33,34,35,36,37,38,39,40,41] are cited in the Supplementary Materials.
