# Supplementary material for: Barriers to and Facilitators of School Health Care for Students with Chronic Disease as Perceived by Their Parents: A Mixed Systematic Review
Source: Healthcare (Basel). 2020 Nov 21;8(4):506. doi: 10.3390/healthcare8040506 (PMC7712821; doi:10.3390/healthcare8040506)
Supplement: Supplementary file 1 [file healthcare-08-00506-s001.pdf]

Supplementary material

Table S1. Critical appraisal of potentially selected articles

Quantitative studies

(*N*=12)

Author and published year	Evaluation items				
	**1**	**2**	**3**	**4**	**5**
Alaqeel (2019) [27]	Y	Y	Y	Y	Y
Driscoll et al. (2015) [37]	Y	Y	Y	Y	Y
Engelke & Swanson (2013) [40]	Y	Y	Y	Y	Y
Herbert et al. (2015) [9]	Y	Y	Y	Y	Y
McCollum et al. (2019) [32]	Y	Y	Y	Y	Y
Mujuru et al. (2011) [31]	Y	Y	Y	Y	Y
Mustafa et al. (2018) [38]	Y	Y	Y	Y	Y
Peery, Engelke, & Swanson (2012) [41]	Y	Y	Y	Y	Y
Särnblad et al. (2014) [35]	Y	Y	Y	Y	Y
Skelley et al. (2013) [36]	Y	Y	Y	Y	Y
Pérez et al. (2019) [29]	Y	Y	Y	Y	Y
Wilt (2020) [39]	Y	Y	Y	Y	Y

Y=Yes

Evaluation items

1. Appropriate sampling strategy
2. Target population
3. Appropriate measurements
4. Low nonresponse bias
5. Appropriate statistical analysis

Qualitative studies

(*N*=6)

Author and published year	Evaluation items				
	**1**	**2**	**3**	**4**	**5**
Al Aloola et al. (2017) [33]	Y	Y	Y	Y	Y
Bechara et al. (2018) [6]	Y	Y	Y	CT	Y
Cheng et al. (2010) [7]	Y	Y	Y	Y	Y
Nieto-Eugenio et al. (2020) [28]	Y	Y	Y	Y	Y
Snieder et al. (2017) [34]	Y	Y	Y	Y	Y
Volerman et al. (2018) [11]	Y	Y	Y	Y	Y

Y=Yes, CT= cannot tell

Evaluation items

1. Appropriate research question
2. Adequate data collection
3. Adequate data analysis
4. Sufficiently substantiated interpretation by data
5. Coherence between data sources, collection, analysis and interpretation

Mixed studies

(*N*=2)

Author and published year	Evaluation items														
	**1**	**2**	**3**	**4**	**5**	**6**	**7**	**8**	**9**	**10**	**11**	**12**	**13**	**14**	**15**
Pinelli et al. (2011) [12]	Y	Y	Y	Y	Y	Y	Y	Y	CT	CT	Y	Y	Y	CT	CT
Rivkina et al. (2014) [30]	Y	Y	Y	Y	Y	Y	Y	Y	CT	Y	Y	Y	Y	CT	Y

Y=Yes, CT= cannot tell
Evaluation items

1. Appropriate sampling strategy
2. Target population
3. Appropriate measurements
4. Low nonresponse bias
5. Appropriate statistical analysis
6. Appropriate research question
7. Adequate data collection
8. Adequate data analysis
9. Sufficiently substantiated interpretation by data
10. Coherence between data sources, collection, analysis and interpretation
11. Adequate rationale for using a mixed method
12. Different components of the study effectively integrated to answer the research question
13. The outputs of the integration of qualitative and quantitative components adequately interpreted
14. Divergences and inconsistencies between quantitative and qualitative results adequately addressed
15. Different components of the study adhere to the quality criteria of each tradition of the methods involved

Table S2. Summary of the included studies

(*N*=20)

**Author (year), Country**	**Design (data collection)**	**Type of students' chronic disease, Number of parent participants, (child's age or grade)**	**Barriers and Facilitators**
**Al Aloola et al. (2017), Saudi Arabia [33]**	Qualitative: Content analysis (Individual Interviews)	Asthma, 19, (8~10 years)	Barriers identified:<br>Interpersonal level: *Limited communication between school and family, difficulties in peer relationship*<br>Institution level: *Unsafe school environment, insufficient* *services for parents*<br>Facilitators identified:<br>Institution level: *Staff-related facilitators, supported self-care, services for parents, tight-knit community*<br>Public and policy level: *Clear action plan and legal support for school health policy*
**Bechara et al. (2018), Brazil [6]**	Qualitative: Content analysis (Individual Interviews)	T1DM, 32, (Primary school age)	Barriers identified:<br>Interpersonal level: *Lack of collaboration with school professionals, difficulties in peer relationship*<br>Institutional-level: *Unsafe school environment, insufficient advocacy*<br>Facilitators identified:<br>Interpersonal level: *School-based intervention*<br>Institutional level*: Safe school environment, services for parents*
**Cheng et al. (2010), Taiwan [7]**	Qualitative: Content analysis (Individual Interview)	Asthma, 15 (7~11 years, Grades 1~3)	Barriers identified:<br>Interpersonal level: *Difficulties in peer relationship*<br>Institutional level: *Unsafe school environment*<br>Facilitators identified:<br>*Collaboration with school professionals and clear role delineation*

**Nieto-Eugenio et al. (2020), Spain [28]**	Qualitative: Grounded theory methods (Individual and dyadic interview)	T1D, food allergy 14, (3~11 years)	Barriers identified:<br>Intrapersonal level: *Lack of knowledge and awareness of school staff*<br>Interpersonal level: *Limited communication between school and family,*<br>Institutional level: *Limited school guidelines, services for parents*<br>Public and policy level: *Limited school policy, lack of coordination between educational and health care system*
**Snieder et al. (2017), USA [34]**	Qualitative: An iterative process (Individual and focus group interviews)	Asthma, 12, (Elementary school age)	Barriers identified:<br>Interpersonal level: *Limited communication between school and family*<br>Facilitators identified:<br>Interpersonal level: *Parental engagement, collaboration with school professionals and clear role delineation, school-based intervention*<br>Institutional level: *Services for parents*
**Volerman et al. (2018), USA [11]**	Qualitative: Grounded theory methods (Focus group interviews)	Asthma, 22, (Elementary and middle school age)	Barriers identified:<br>Intrapersonal level: *Lack of knowledge and awareness of school staff*<br>Interpersonal level*: Lack of collaboration with school professionals*<br>Institutional level: *Limited school guidelines, limited self-care support*<br>Public and policy level: *Limited school policy*<br>Facilitators identified:<br>Intrapersonal level: *Action plan*<br>Interpersonal level: *Effective communication between school and family parental engagement*<br>Institutional level: *School staff-related facilitators, supported self-care, tight-knit community*<br>Public and policy level: *Clear action plan and legal support for school health policy*
**Alaqeel (2019), Saudi Arabia [27]**	Quantitative: Descriptive study (Survey)	T1DM, 411, (3~19 years)	Barriers identified:<br>Intrapersonal level: *Lack of knowledge and awareness of school staff*<br>Institutional level: *Limited school guidelines, school staff-related barriers, unsafe school environment*<br>Public and policy level: *Lack of coordination between educational and health care system, inequities of health care and inaccessibility to school*
**Driscoll et al. (2015), USA [37]**	Quantitative: Descriptive study (Survey)	T1DM, 435, (5~17 years)	Barriers identified:<br>Institutional level: *School staff-related barriers, unsafe school environment*<br>Facilitators identified:<br>Institutional level: *School staff-related facilitators*
**Engelke & Swanson (2013), USA [40]**	Quantitative: Descriptive study (Survey)	Asthma, 134, (Grades 1~12)	Facilitators identified:<br>Institutional level: *Supported self-care*
**Herbert et al. (2015), USA [9]**	Quantitative: Descriptive study (Survey and medical chart review)	T1DM, 134, (Mean 5.3 years)	Barriers identified:<br>Institutional level: *School staff-related barriers, unsafe school environment*
**McCollum et al. (2019), Ireland [32]**	Quantitative: Descriptive study (Survey)	T1DM, 418 (4~13 years)	Barriers identified:<br>Intrapersonal level: *Lack of parental knowledge*<br>Institutional level: *School staff-related barriers*<br>Public and policy level*: Inequity of school health care and inaccessibility to school*<br>Facilitators identified:<br>Interpersonal level: *Parental engagement*
**Mujuru et al. (2011), USA [31]**	Quantitative: Descriptive study (Survey)	Asthma, 13, (Grades 3~5)	Barriers identified:<br>Intrapersonal level: *Lack of parental knowledge*<br>Facilitators identified:<br>*Intrapersonal level: School-based intervention*<br>Institutional level: *Supported self-care*
**Mustafa et al. (2018), USA [38]**	Quantitative: Descriptive study (Online survey)	Food allergy, 289, (Grades 1~12)	Barriers identified:<br>Institutional level: *School staff-related barriers, unsafe school environment, limited self-care support*<br>Public and policy level: *Limited school policy, inequities of school health care and inaccessibility to school*

**Peery, Engelke, & Swanson (2012), USA [41]**	Quantitative: Descriptive study (Survey)	Diabetes, 69, (Elementary, middle, and high school age)	Facilitators identified:<br>Institutional level: *Supported self-care*
**Särnblad et al. (2014) Sweden [35]**	Quantitative: Descriptive study (Survey)	T1DM, 323, (Mean 11.4 years)	Barriers identified:<br>Institutional level: *Unsafe school environment*<br>Facilitators identified:<br>Interpersonal level: *Parental engagement*<br>Institutional level: *School staff-related facilitators*<br>Public and policy level: *Clear action plan and legal support for school health policy*
**Skelley et al. (2013), USA [36]**	Quantitative: Descriptive study (Survey)	Diabetes, 148, (Mean 12.5, 11.9 years)	Barriers identified:<br>Institutional level: *School staff-related barriers*, *unsafe school environment*<br>Public and policy level: *Inequity of school health care and inaccessibility to school*<br>Facilitators identified:<br>Institutional level: *School staff-related facilitators*, *safe school environment*
**Pérez et al. (2019), Spain [29]**	Quantitative: Descriptive study (Survey)	T1DM, 206, (3~16 years)	Barriers identified:<br>Intrapersonal level: *Lack of knowledge and awareness of school staff, lack of parental knowledge*<br>Institutional level: *School staff-related barriers*, *unsafe school environment*, *services for parents*
**Wilt (2020), USA [39]**	Quantitative: Descriptive study (Survey)	T1DM, 89, (10~16 years)	Barriers identified:<br>Institutional level: *Insufficient services for parents*<br>Facilitators identified:<br>Institutional level: *School staff-related facilitators*, *supported self-care*
**Pinelli et al. (2011), Australia [12]**	Mixed methods: mechanical and interpretative analysis, and descriptive study (Focus group interview and survey)	T1DM, 220 for survey, Not applicable, for interview, (6~13 years)	Barriers identified:<br>Intrapersonal level: *Lack of knowledge and awareness of school staff*<br>Institutional level: *School staff-related barriers*, *unsafe school environment*<br>Facilitators identified:<br>Interpersonal level: *Parental engagement*
**Rivkina et al. (2014), USA [30]**	Mixed methods: Content analysis and descriptive study (Focus group and key informant interview, and survey)	Asthma, food allergy, 72, (School age)	Barriers identified:<br>Intrapersonal level: *Lack of parental knowledge*<br>Interpersonal level: *Limited communication between school and family*<br>Institutional level: *School staff-related barriers*
